# Supplementary material for: Iterative improvement in the automatic modular design of robot swarms
Source: PeerJ Comput Sci. 2020 Dec 7;6:e322. doi: 10.7717/peerj-cs.322 (PMC7924708; doi:10.7717/peerj-cs.322)
Supplement: Supplemental Information 3 [file peerj-cs-06-322-s003.zip › argos3/doc/api/standalone/a00408_source.html]

ARGoS: core/wrappers/lua/lua\_controller.cpp Source File


- Main Page
- Related Pages
- Namespaces
- Classes
- Files

- File List
- File Members

# core/wrappers/lua/lua\_controller.cpp

Go to the documentation of this file.

```
00001 
00007 #include "lua_controller.h"
00008 #include <argos3/core/utility/logging/argos_log.h>
00009 
00010 #include <argos3/core/wrappers/lua/lua_utility.h>
00011 
00012 namespace argos {
00013 
00014    /****************************************/
00015    /****************************************/
00016 
00017    CLuaController::CLuaController() :
00018       m_ptLuaState(NULL),
00019       m_bScriptActive(false),
00020       m_bIsOK(true),
00021       m_pcRNG(NULL) {
00022    }
00023 
00024    /****************************************/
00025    /****************************************/
00026 
00027    CLuaController::~CLuaController() {
00028    }
00029 
00030    /****************************************/
00031    /****************************************/
00032 
00033    void CLuaController::Init(TConfigurationNode& t_tree) {
00034       try {
00035          /* Create RNG */
00036          m_pcRNG = CRandom::CreateRNG("argos");
00037          /* Load script */
00038          std::string strScriptFileName;
00039          GetNodeAttributeOrDefault(t_tree, "script", strScriptFileName, strScriptFileName);
00040          if(strScriptFileName != "") {
00041             SetLuaScript(strScriptFileName, t_tree);
00042             if(! m_bIsOK) {
00043                THROW_ARGOSEXCEPTION("Error loading Lua script \"" << strScriptFileName << "\": " << lua_tostring(m_ptLuaState, -1));
00044             }
00045          }
00046          else {
00047             /* Create a new Lua stack */
00048             m_ptLuaState = luaL_newstate();
00049             /* Load the Lua libraries */
00050             luaL_openlibs(m_ptLuaState);
00051             /* Create and set Lua state */
00052             CreateLuaState();
00053             SensorReadingsToLuaState();
00054          }
00055       }
00056       catch(CARGoSException& ex) {
00057          THROW_ARGOSEXCEPTION_NESTED("Error initializing Lua controller", ex);
00058       }
00059    }
00060 
00061    /****************************************/
00062    /****************************************/
00063 
00064    void CLuaController::ControlStep() {
00065       if(m_bScriptActive && m_bIsOK) {
00066          /* Update Lua state through sensor readings */
00067          SensorReadingsToLuaState();
00068          /* Execute script step function */
00069          if(! CLuaUtility::CallLuaFunction(m_ptLuaState, "step")) {
00070             m_bIsOK = false;
00071          }
00072       }
00073    }
00074 
00075    /****************************************/
00076    /****************************************/
00077 
00078    void CLuaController::Reset() {
00079       if(m_bScriptActive) {
00080          if(m_bIsOK) {
00081             m_bIsOK = CLuaUtility::CallLuaFunction(m_ptLuaState, "reset");
00082          }
00083          else {
00084             SetLuaScript(m_strScriptFileName);
00085          }
00086       }
00087    }
00088 
00089    /****************************************/
00090    /****************************************/
00091 
00092    void CLuaController::Destroy() {
00093       if(m_bScriptActive && m_bIsOK) {
00094          /* Execute script destroy function */
00095          CLuaUtility::CallLuaFunction(m_ptLuaState, "destroy");
00096       }
00097       /* Close Lua */
00098       lua_close(m_ptLuaState);
00099    }
00100 
00101    /****************************************/
00102    /****************************************/
00103 
00104    void CLuaController::SetLuaScript(const std::string& str_script,
00105                                      TConfigurationNode& t_tree) {
00106       /* First, delete old script */
00107       if(m_bScriptActive) {
00108          lua_close(m_ptLuaState);
00109          m_bScriptActive = false;
00110          m_strScriptFileName = "";
00111       }
00112       /* Create a new Lua stack */
00113       m_ptLuaState = luaL_newstate();
00114       /* Load the Lua libraries */
00115       luaL_openlibs(m_ptLuaState);
00116       /* Load script */
00117       if(!CLuaUtility::LoadScript(m_ptLuaState, str_script)) {
00118          m_bIsOK = false;
00119          return;
00120       }
00121       m_strScriptFileName = str_script;
00122       /* Create and set variables */
00123       CreateLuaState();
00124       SensorReadingsToLuaState();
00125       ParametersToLuaState(t_tree);
00126       /* Execute script init function */
00127       if(!CLuaUtility::CallLuaFunction(m_ptLuaState, "init")) {
00128          m_bIsOK = false;
00129          return;
00130       }
00131       m_bIsOK = true;
00132       m_bScriptActive = true;
00133    }
00134 
00135    /****************************************/
00136    /****************************************/
00137 
00138    void CLuaController::SetLuaScript(const std::string& str_script) {
00139       TConfigurationNode t_tree;
00140       SetLuaScript(str_script, t_tree);
00141    }
00142    
00143    /****************************************/
00144    /****************************************/
00145 
00146    void CLuaController::CreateLuaState() {
00147       /* Register functions */
00148       CLuaUtility::RegisterLoggerWrapper(m_ptLuaState);
00149       /* Create a table that will contain the state of the robot */
00150       lua_newtable(m_ptLuaState);
00151       /* Set the id of the robot */
00152       lua_pushstring(m_ptLuaState, "id");
00153       lua_pushstring(m_ptLuaState, GetId().c_str());
00154       lua_settable(m_ptLuaState, -3);
00155       /* Register RNG */
00156       CLuaUtility::RegisterRNG(m_ptLuaState, m_pcRNG);
00157       /* Go through devices and add the necessary items to the table */
00158       for(CCI_Actuator::TMap::iterator it = m_mapActuators.begin();
00159           it != m_mapActuators.end();
00160           ++it) {
00161          it->second->CreateLuaState(m_ptLuaState);
00162       }
00163       for(CCI_Sensor::TMap::iterator it = m_mapSensors.begin();
00164           it != m_mapSensors.end();
00165           ++it) {
00166          it->second->CreateLuaState(m_ptLuaState);
00167       }
00168       /* Set the name of the table */
00169       lua_setglobal(m_ptLuaState, "robot");
00170    }
00171 
00172    /****************************************/
00173    /****************************************/
00174 
00175    void CLuaController::SensorReadingsToLuaState() {
00176       /* Put the robot state table on top */
00177       lua_getglobal(m_ptLuaState, "robot");
00178       /* Go through the sensors */
00179       for(CCI_Sensor::TMap::iterator it = m_mapSensors.begin();
00180           it != m_mapSensors.end();
00181           ++it) {
00182          it->second->ReadingsToLuaState(m_ptLuaState);
00183       }
00184       /* Pop the robot state table */
00185       lua_pop(m_ptLuaState, 1);
00186    }
00187 
00188    /****************************************/
00189    /****************************************/
00190 
00191    void CLuaController::ParametersToLuaState(TConfigurationNode& t_tree){
00192       /* Put the robot state table on top */
00193       lua_getglobal(m_ptLuaState, "robot");
00194       /* Add a table for the XML params to the global symbol table and fill it with values */
00195       CLuaUtility::OpenRobotStateTable(m_ptLuaState, "params");
00196       TConfigurationAttributeIterator it;
00197       std::string strKey;
00198       std::string strValue;
00199       for (it = it.begin(&t_tree); it != it.end(); ++it) {
00200          it.Get()->GetName(&strKey);
00201          it.Get()->GetValue(&strValue);
00202          CLuaUtility::AddToTable(m_ptLuaState, strKey, strValue);
00203       }
00204       CLuaUtility::CloseRobotStateTable(m_ptLuaState);
00205       /* Pop the robot state table */
00206       lua_pop(m_ptLuaState, 1);
00207    }
00208 
00209    /****************************************/
00210    /****************************************/
00211 
00212    std::string CLuaController::GetErrorMessage() {
00213       if(m_bIsOK) {
00214          return "OK";
00215       }
00216       else {
00217          SInt32 i = 1;
00218          while(i <= lua_gettop(m_ptLuaState) && lua_type(m_ptLuaState, i) != LUA_TSTRING) {
00219             CLuaUtility::PrintStack(LOGERR, m_ptLuaState);
00220             ++i;
00221          }
00222          if(i > lua_gettop(m_ptLuaState)) return "Unknown compilation error";
00223          else return lua_tostring(m_ptLuaState, i);
00224       }
00225    }
00226 
00227    /****************************************/
00228    /****************************************/
00229 
00230    REGISTER_CONTROLLER(CLuaController, "lua_controller");
00231 
00232 }
```

---

Generated on 10 Jul 2018 for ARGoS by 
 1.6.1
